# Proposal of a population wide genome-based testing for Covid-19

**DOI:** 10.1038/s41598-022-08934-2

**Published:** 2022-04-04

**Authors:** Hans Lehrach, Jon Curtis, Bodo Lange, Lesley A. Ogilvie, Richard Gauss, Christoph Steininger, Erhard Scholz, Matthias Kreck

**Affiliations:** 1 Alacris Theranostics GmbH, Berlin, Germany; 2 Max Planck Institute for Molecular Genetics, Ihnestraße 63, 14195 Berlin, Germany; 3 State Sanitary Directorate, City Government, Vienna, Austria; 4 Department of Medicine I, Division of Infectious Diseases, Medical University of Vienna, Vienna, Austria; 5 LEAD Horizon, Vienna, Austria; 6 University of Wuppertal, Wuppertal, Germany; 7 University of Bonn, Bonn, Germany; 8 University of Frankfurt, Frankfurt, Germany

**Keywords:** Computational models, Microbiology, SARS-CoV-2, PCR-based techniques

## Abstract

Our lives (and deaths) have by now been dominated for two years by COVID-19, a pandemic that has caused hundreds of millions of disease cases, millions of deaths, trillions in economic costs, and major restrictions on our freedom. Here we suggest a novel tool for controlling the COVID-19 pandemic. The key element is a method for a population-scale PCR-based testing, applied on a systematic and repeated basis. For this we have developed a low cost, highly sensitive virus-genome-based test. Using Germany as an example, we demonstrate by using a mathematical model, how useful this strategy could have been in controlling the pandemic. We show using real-world examples how this might be implemented on a mass scale and discuss the feasibility of this approach.

## Introduction

To combat the spread of COVID-19, most countries have relied on social distancing, lockdown, limited testing and contact tracing procedures, as well as crash programs to develop vaccines, which are now increasingly contributing to the fight against the pandemic. As a complement to these two basic strategies, we and others proposed to use an alternative method—repeated population-wide tests^1–3^, to identify (and quarantine) the infected and break infection chains. This strategy has the potential to reduce human suffering, economic costs and restrictions on personal freedom to the inevitable minimum, since quarantine would be limited to a short time for a small fraction of the population and could be much more effective than inherently leaky lockdowns.

Through this, we could have controlled (and, in combination with contact tracing or longer term lower level testing, potentially eliminated) the pandemic safely, quickly and comparatively inexpensively regionally, nationally and possibly worldwide. This raises the question why this strategy, to our knowledge, has never been seriously evaluated as an alternative and complement to the (not uniformly successful) approaches, which have been followed.

The benefits and challenges of one of the most potentially powerful strategies in our arsenal against COVID-19 have been debated widely^4–6^, fuelled by the varied results gleamed from population-scale pilots^7–11^. However, many of these pilots have often been restricted to a single test cycle and/or relied on rapid antigen tests, which are inherently limited in their ability to stop new infections^7–10^. Despite the existence of successful examples, (e.g. Vò, an Italian city with 3300 inhabitants^11^), few governments seem to have seriously considered this as an alternative and, with the exception of Austria (‘Alles gurgelt’)^12^, none seem to have supported the establishment of the required infrastructure.

We can only speculate why the strategy of low cost and highly sensitive population wide tests was not even considered. If doubts concerning the feasibility is the main reason, we hope that this paper is a reason to consider our approach seriously.

To counteract these arguments, we describe here the successful implementation of the two main elements needed: sensitive, scalable and cost-effective PCR based tests and a logistic infrastructure for population-wide tests. Using mathematical modelling, the enormous impact this strategy could have had on the pandemic in Germany is demonstrated; a country still dealing with the brutal consequences of COVID-19, with over 110,000 deaths, hundreds of billions of euros in economic costs and continuing restrictions on social freedoms for all. The overwhelming majority of this impact, including close to 90% of deaths, was caused by the second, third and now fourth waves of the pandemic, and could therefore have been potentially avoided by PCR-based mass testing.

Although there are a variety of studies concerning epidemiological evaluation and theoretical modelling of the effect of mass testing^13–15^, the novelty of the current work is an approach for mass testing and the modelling of its potential impact using a new mathematical model adjusted to COVID-19.

## Results

### An affordable, scalable and highly sensitive virus-genome-based testing approach

A key component of the proposed strategy has been the development of highly-scalable, cost effective techniques to detect the viral genome with high sensitivity and specificity before infected persons become infectious themselves, in millions and ultimately billions of samples. This basically rules out the use of antigen-based rapid tests, which typically miss the first three days of a ten-day-long infectious period^16,17^. Standard qPCR tests, with their ability to identify individual genome molecules would be sufficiently sensitive: a new infection can only take place after an eclipse phase, which ends sometime after detectable RNA concentrations have been reached^18^. They are, however, not sufficiently scalable (e.g. Germany’s test capacity is currently (January 2022) approx. 370,000 tests per day^19^) and the cost would be prohibitive.

**Figure 1:**
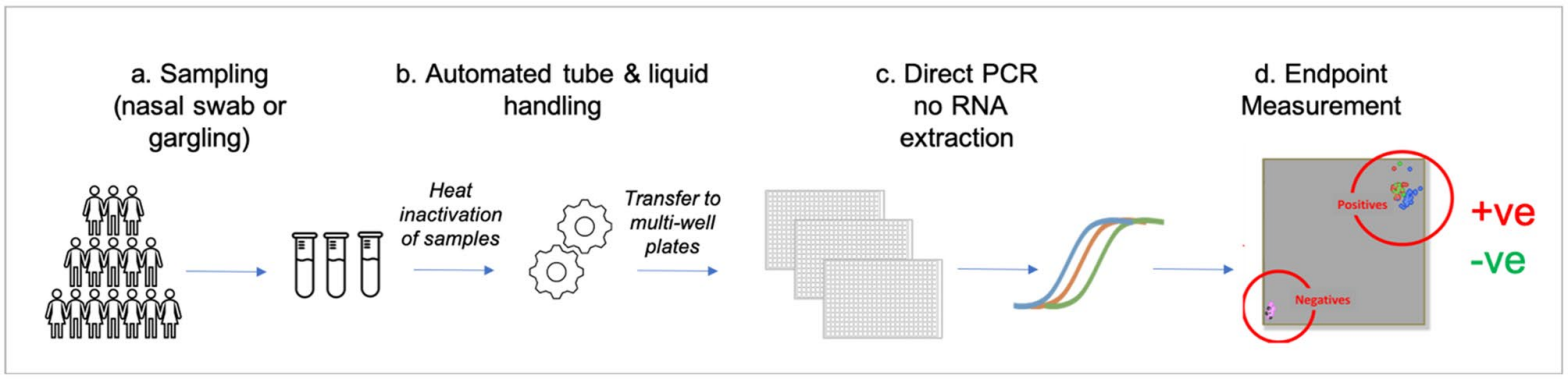
Schematic of the high throughput genome -based testing pipeline for SARS-CoV-2. (**a**) Sample tubes (barcoded) are brought to testing centres in 96-well carriers from collection points (e.g. see Fig. 2) and prepared for high-throughput testing for SARS-CoV-2 (or other viruses). Samples are heat inactivated before automated transfer in 384-well format (**b**) and preparation for (**c**) direct RT-PCR without a requirement for RNA extractions and (**d**) endpoint measurement showing results of test (positive, negative).

We have therefore developed a ×10–×20 more efficient, highly sensitive virus-genome-based test, using PCR technologies^3,20–22^ developed by some of us as part of the Human Genome Project^20^, and used to genotype billions of samples over the last decades^3^. All equipment is commercially available. Since we use the same reactions as standard qPCR tests but read the result differently (endpoint measurement^3,21,22^), we achieve the same sensitivity and specificity in a much more scalable fashion and at much lower costs per sample (~ € 1 per PCR test for very high throughput). See Fig. 1 for overview of the test pipeline.

The procedure is EU-wide CE approved in gargle- and smear-based versions and has already been used in Germany to test the employees of Unilever and other companies. The key advantage is that infected individuals can be identified days earlier than with rapid antigen tests^17,18^ currently used by the vast majority of companies; a prerequisite to stopping the spread of the virus. The test is highly scalable: A single commercially available water bath PCR system with a capacity of 100 × 384 PCR plates per run would be able to carry out > 600,000 RT-PCR reactions per day (significantly more than the daily PCR test capacity in Germany as of January 2022^19^). A potential limitation is still the supply of test tubes and pipette tips (one per sample, far less than for standard qPCR tests). However, equipment to wash and reuse pipette tips is commercially available and approved for COVID-19^23,24^ and machines to reuse the sample tubes could be rapidly developed based on similar principles. The approach can also, in a slightly modified form, be easily used to identify sequence variants^25^. We would therefore be able to identify the most relevant groups of variants (e.g. B.1.1.7, alpha variant; B.1.351, beta variant; P.1, gamma variant; P.2, zeta variant; B.1.617.1, kappa; B.1.617.2, delta; and the most recent omicron variant B.1.1.529) in a second analysis cycle with a small number of additional tests on SARS-CoV-2 positive samples.

### Logistics: population-scale proof-of-concept

**Figure 2:**
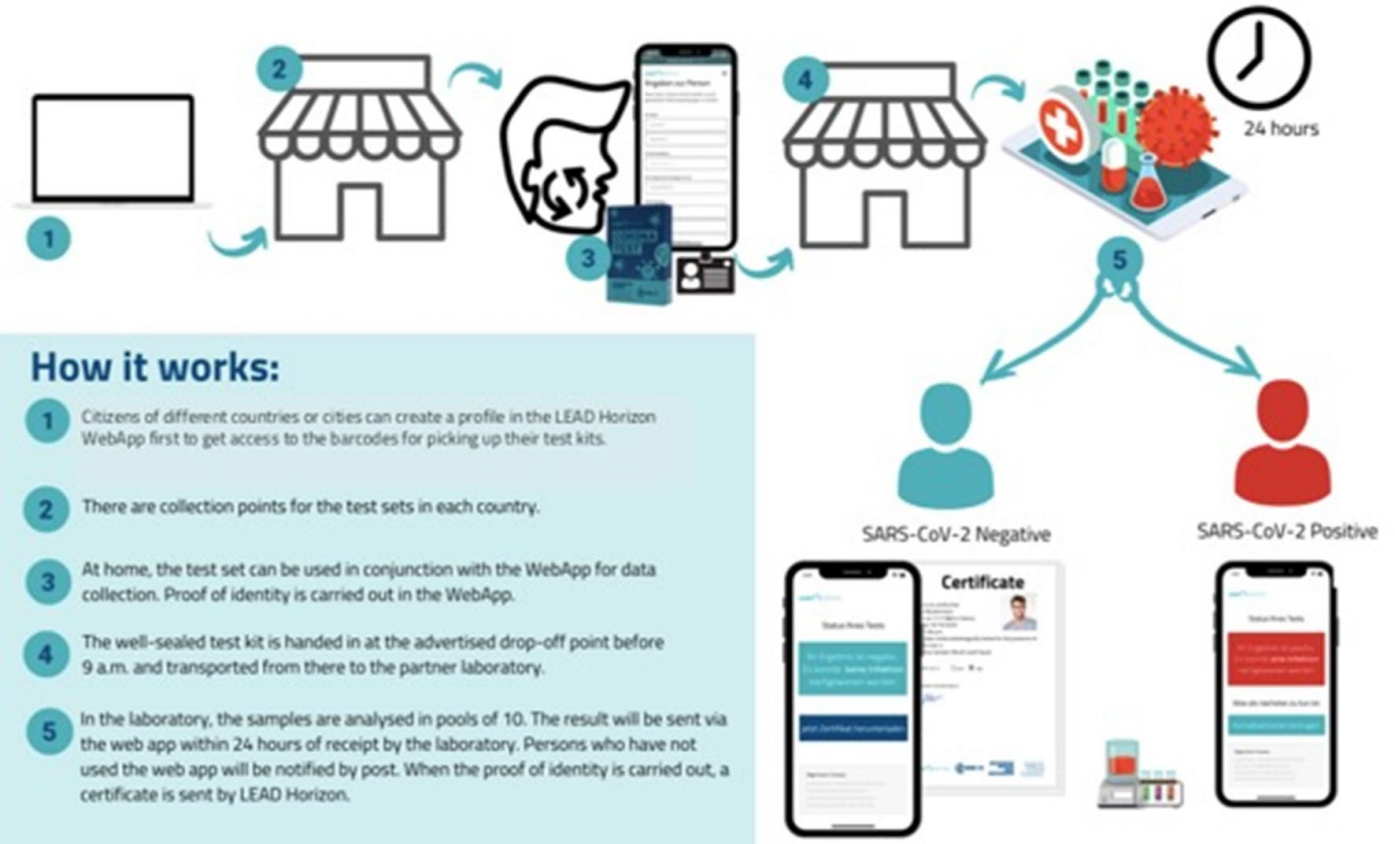
Population-wide testing logistics. An example of the decentralized test strategy used in Vienna.

As a proof-of-concept, the government of Vienna, Austria, established a large, state-wide screening program which demonstrates the feasibility of such an approach (‘Alles gurgelt’)^12^. The aim of this program is the early interruption of infection chains, as well as tracing and prevention of spread of SARS CoV-2. For this purpose, a novel logistics concept was developed based on self-collected mouth wash samples. Self-collection of samples is done with support of a dedicated Web App, which guides the user through the procedure and validates their identity by verification of an identity card and video-surveillance of the procedure (www.lead-horizon.com), so that quality and reproducibility of sample-taking can be ensured. The testing program is based on the promise that a reliable PCR test result is available for every inhabitant within a maximum of 24 h after sample collection. All inhabitants were invited to participate in this program twice a week as regular testing makes sure that persons who have tested positive for COVID-19 can be quarantined and chains of infection interrupted early. Test kits for the validated self-collection of mouth-wash samples are distributed through local drug stores, which makes PCR tests available to all inhabitants within reach of a 5-min walk. Following packaging in biohazard safe, sealed transport bags and cardboard packaging, samples are returned for transport to the laboratory at drug and grocery stores in Vienna. Sampling devices are made accessible free of charge to all people living in Vienna, tourists and commuters. The successful program is currently being expanded in more pilot regions of Austria for further evaluation of a full, nation-wide coverage of the program. As an alternative, a similar kit based on nasal swabs, which might be easier to interface with the high throughput testing pipeline described here, has been developed at Alacris Theranostics. See Fig. 2 for an outline of how the population-scale testing works in practice.

### Modelling the impact of population-scale testing in Germany

To model the impact of repetitive population wide (or near-population-wide) testing, we show the effect this approach could have had on the development of the pandemic in Germany if population-wide tests were started last autumn, using a model adjusted to COVID-19. The model is a discrete version of the Kermack–McKendrick theory and has a central input: A function ɣ measuring how infectious a person is k days after getting infected. This curve depends on the virus and is rescaled by a higher factor for more aggressive mutants. This function together with information about the contacts determines the model for asymptomatic people. For those sent to quarantine the function is set to 0 from that day on. So the model is very sensitive to how quickly infected persons can be sent into quarantine. Besides the contact rate, the effect of vaccinations is implemented into the model by reducing the numbers of susceptible people. Information about the function ɣ is derived from virological as well as from epidemiological studies. Information about contact rates is derived from empirical data applied to the model. Information about the asymptomatic people is derived from virological studies, and vaccination from data available from the Robert Koch Institute (RKI). This and the choice of the central function ɣ is carefully carried out in section 3 in Kreck and Scholz^26^ (see sources therein). We discuss the derivation of the ɣ function from three different perspectives, virological data, epidemiological data and a set of key data (duration of the exposed period, the occurrence of the peak and the duration of the infectious period) which allow to establish the function using mathematical tools. All three perspectives lead to approximately equal functions, from which we chose one which has a very high plausibility. Programming the model is self-explanatory since it is a recursive model.

**Figure 3:**
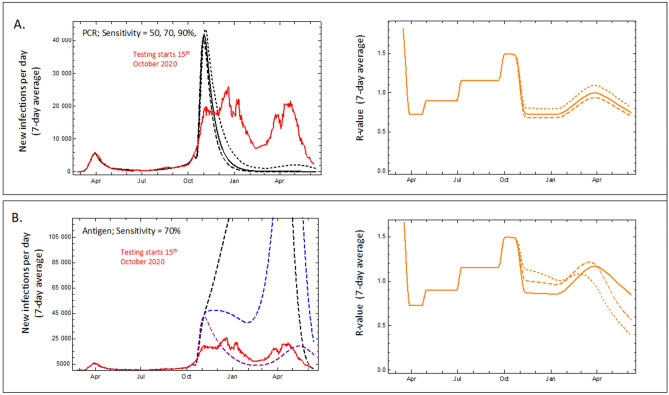
Modelling the impact of population-scale testing in Germany. Graphs show the comparison of the development of registered daily new infections (7-day averages) of John Hopkins University data for Germany (red line) with model values. The model assumes daily testing starting Oct 15 2021, gradually increasing participation up to 60% of the population after 30 days. (**A**) (PCR) daily testing of 60% of the population with sensitivity 50% (dashed), 70% (solid line), 90% (dotted). Left: daily registered newly infected. Right: R-value. (**B**) (Antigen) daily testing of 60% of the population with sensitivity 70% under the assumption of test effectiveness delayed with respect to PCR tests by 1, 2 or 3 days. Left: expected daily new infections with delay by 1 day (purple), 2 days (blue), respectively 3 days (black). Right: corresponding R-values.

To simplify the analysis, it is assumed that tests are 100% reliable and a certain percentage of the population is tested on a daily basis (see Kreck and Scholz^26^). Depending on how large this percentage is, one can predict what would have happened in Germany if such tests would have started on October 15th 2020; a realistic scenario, since there would have been enough time to develop the required technology and infrastructure, if these developments had been supported by the government or private sources. In Fig. 3, the numbers of daily new registered infected are displayed and corresponding R-values are shown, under the assumption that 60%, 80% or 100% of the population agreed to be tested on a daily basis using PCR and antigen-based tests, respectively.

Assumptions and consequences of the scenarios:

- PCR tests are effective the day people get infectious. Depending on how carefully samples are taken the sensitivity may differ^27^. To demonstrate the effect of this we model three cases, sensitivity 50%, 70% or 90%. Surprisingly the influence is not large. False positives are ignored for the model calculation (corresponding to a hypothetical specificity of 100%). Model contact rates are evaluated from the empirical data (Johns Hopkins University & Medical, Coronavirus Resource Centre^28^) until October 15, 2020. They are then kept fixed for the model calculations of the tests. In reality, further strong restrictions were imposed in Germany after this date. The model scenarios allow evaluation of whether such further restrictions would have been necessary to keep the dynamics under control.
- Vaccinations are incorporated in the model calculation from January 2021 onwards according to German data (https://impfdashboard.de). The B.1.1.7 alpha variant started to spread in Germany at the end of 2020. According to the data of the RKI, 2% of all sequenced samples were identified as variant of concern B1.1.7 in the first calendar week (CW); in early April (CW 14) its rate had risen to 88.1% and was dominant until the rise of the delta variant^29^.
- Rapid antigen tests vary drastically in their sensitivity, as a recent study of the Paul Ehrlich Institute demonstrates^30^. For that reason, it is impossible to design a realistic scenario for their application analogous to the one here proposed. However, even a hypothetical comparison of tests which are effective only at day 2, or 3 or 4 of the period of infectivity is useful (Fig. 3). The comparisons illustrate the effects of time delays to be expected for antigen tests. A recent study indicates a delay between two and four days for sensitivity of antigen tests for the omicron variant^17^. Apart from this difference, we make the same assumptions as for PCR tests. The surprising result is that the tests that are already effective at day two of the period of infectivity generate much worse results than PCR tests, and for those tests effective at day three or four, the difference is drastic. We view this as the main message from our model scenarios.

If the sensitivity of PCR tests falls below 90% (sensitivity of 50%, 70% or 90% was assumed for the model calculation) the picture does not change qualitatively. A specificity of 100% may be assumed for the model, because the specificity of PCR reactions is very high (two specific primers, one specific probe), and can be further increased by, e.g. running a second cycle of analysis to identify the specific variant. Technical errors (cross-contamination of samples etc.) can be excluded by appropriate controls. In the calculation, effective enforcement of quarantine rules is assumed. Low levels of non-compliance would effectively decrease the overall participation rate, without major effect on the result. The model calculations therefore demonstrate convincingly the effectiveness of genome-based mass tests under only moderate contact restrictions like those in Germany in early October 2020. Even the rise of the new alpha virus variant in early 2021 could have been kept under control by mass testing using highly sensitive PCR based tests, suppressing the R factor from 1.5 before the onset of testing to very low levels for a high degree of participation. Even low levels of participation (60%) are sufficient to reach R values far below 1, and therefore to suppress the pandemic. This is not the case at all if we model the effect of the rapid antigen tests, which are less sensitive^29,30^, but also, more importantly, only become positive after onset of symptoms fairly late in the infectious phase^17^. In this case, only 100% participation in daily testing would have reduced the R factor slightly below 1, only to return to values above 1, when the alpha variant arrived. The drop in new infections shown in Fig. 3 for the 80% and 60% scenarios would have been due to ‘herd immunity’ after very high levels of infection. While useful for other applications, tests that are effective only at day two, three or four of the infectious period, which seems to be the case for rapid antigen tests^17^ can therefore not be used successfully in this particular strategy.

## Discussion

The question of whether the strategy that has been applied in almost all countries, namely to restrict the lives of the whole population, is the best way to fight the COVID-19 pandemic is of very high relevance. In all democratic countries the rights of individuals are the highest value and central in all constitutions. To restrict these rights drastically is only justified if there are no alternative ways to control the pandemic.

Scientific advisory boards in most countries have argued that there is no alternative to the strategy of social restrictions. We claim that mass testing of large part of the population gives a tool which enables the reduction of social restrictions, and so the deep intrusion into the rights of individuals might be much milder than it was in the last two years. In addition, testing a large enough fraction of the population often enough (both dependent on the infectiosity of the virus, remaining protection provided by vaccination, social restrictions) will allow us to reduce the all-important R factor below one, and therefore, over time, reduce the infection level to a low level reflecting mostly the rate, at which new infections are introduced from the outside.

The main advantage of our strategy is that strong restrictions are only imposed on those who are carrying the virus. For them it is even an advantage since in addition to isolating them from others medical care can be given early enough that the chances of a milder disease course are larger.

The application of mass testing for controlling the pandemic is not a new idea. But since the capacity of PCR tests has been comparatively low and costs high, rapid antigen tests have been mainly used to date, despite reported high variations in sensitivity and varied outcomes in pilot scenarios^7–11,29,30^. Our results contrast with work that argues for the use of rapid antigen tests as part of overall testing strategies^31^, due to two critical differences: (1) We address the described drawbacks of using PCR tests in a population-scale scenario (high cost, limited capacity, delays in returning the result) by our new cost effective and highly scalable PCR tests (endpoint measurement) and (2) In our view the importance of the late response of antigen rapid tests during the infectious period is underestimated.

Our approach is not free of limitations. The modelling carried out here is based on three strong assumptions: (a) To test 60% of the German population every day would require a test capacity of minimally 50 million tests per day (b) To build up an infrastructure which can manage to distribute this number of test kits, to collect samples and deliver them to laboratories, in a similar manner as the logistics used in the 'Alles Gurgelt' project in Vienna (but on a larger scale) and (c) To convince a significant fraction of the population to self-test every day and drop off their samples at collections points. Whether this can be achieved is unclear. Point (a) and (b) will obviously depend on government funding (but so does vaccination). Point (c) might already be achievable by reactivating the regulations governing many activities at the beginning of 2021, e.g. requirement to have a negative test result (in this case obviously PCR-based) for a wide range of activities and/or an appeal to the self-interest of the population (particularly, if early detection of the infection helps to reduce the medical risks due to earlier onset of therapy). It is, however, important to keep in mind that the conditions analysed here, selected to some extent to be easy to model using the models we used, are examples to illustrate the feasibility of the approach. Any testing with quarantine of the positives will reduce the R factor, and many alternative high throughput PCR testing strategies could be used to reduce R below 1, and suppress the pandemic.

On a more technical level, sensitivity of PCR tests will undoubtedly vary dependent on a range of factors such as the variant being detected and the way specimens are collected^27,32^, which would all have to be considered in developing a population-wide programme. However, our modelling results reinforce the effectiveness of the PCR-based testing approach even at sensitivities of 50%. Moreover, even if the most sensitive antigen tests would be applied, our model scenarios highlight that due to the reported later response of antigen tests^17,18^ compared to PCR tests, the former may be less useful for such a strategy.

The pandemic is, however, far from over. Roll-out of vaccinations in many areas of the world has been much slower than originally anticipated (e.g. in India) and new variants threaten to undo the progress made^33^. We should therefore guard against the possibility that new variants could also break the immunity generated by vaccines. As a complement to the effort on vaccination, and an alternative to (inherently risky) endemisation strategies, we should therefore urgently set up (and use) an infrastructure for virus genome-based mass tests to protect the population from increasingly aggressive SARS-CoV-2 variants. Such an infrastructure would also be able to respond to any new, similar threats (essentially all pathogens have genomes) within a few weeks rather than the year(s) new vaccine development is likely to take.

The question what will happen in the future is at its best an open question. Most experts see the danger that either a new mutant will emerge or the currently dominant omicron variant will continue to spread. The protection by being recovered or vaccinated lasts much shorter than originally assumed^34,35^. Given this background, our approach is of high actual relevance. Open discussion of all tools available to tackle the global challenge of COVID-19 is required, including the cost effective and feasible solution presented here.

We have the tools. We just have to be willing to use them.

## Conclusions

We have proposed a strategy of how to apply mass testing of large parts of a population to control the COVID-19 pandemic. The central input is a highly sensitive, cost effective and scalable PCR-based testing strategy (endpoint measurement). We have proposed a strategy of how to establish the procedure of such mass tests effectively. Whether such a procedure can successfully control the pandemic can be studied by a mathematical model which is specifically designed for COVID-19. The result of this modelling shows that even if only 60% of a population takes part in daily tests the pandemic is under control within two or three months—even if the PCR test sensitivity is a low as 50%. There are other tests with such a sensitivity, for example high quality rapid antigen tests. This raises the question whether such tests could be used instead. The key advantage of the PCR tests described here is that they detect the virus very early, even on the first day of the infectious period, whereas other tests are only effective later in the cycle. The somewhat surprising result of our modelling is that tests which are effective only one day later, lead to an insufficient control of infection numbers. Tests which are effective only at day three or four of the infectious period are essentially useless for this approach. All this is demonstrated in a hypothetical scenario where we assume that such tests are applied in October 2020 in Germany. The model shows that with the PCR mass tests control of infection numbers to levels manageable by contact tracing can be achieved, although it is assumed that in contrast to the real developments no further social restrictions are assumed—in contrast to what happened in Germany in November and December.

## Methods

### High-throughput PCR testing

High-throughput PCR based tests are performed on either nasal swabs or gargle samples, collected, if possible, in kits, useful for direct testing and eliminating the need for RNA purification. Samples are received in triple coded screwcap tubes in SBS carriers with 96 positions (including controls). After virus inactivation (as part of transport medium in the case of nasal swabs, by heat treatment for 30 min at 65 °C for gargle samples), the bottom barcodes are read (FluidX Perception HD Whole Rack Reader), the tubes are decapped using an 8 or 96 position decapper (e.g. FluidX XDC-96 Automatic 96-Format Tube Rack Decapper/Capper), and 4 µl (10 µl reactions) or 8 µl (20 µl reactions) are transferred (e.g. Biomek i7 Automated Workstation) using a 96 pipetting head into 384-well PCR plates already containing the appropriate master and assay mixes.

Plates are heat-sealed (e.g. PX1 PCR Plate sealer), and the contents are amplified by RT-PCR reactions using either standard high-throughput PCR machines (e.g. DNA Engine Tetrade 2 Thermal Cycler) or, for very high throughput water bath PCR system (e.g. KBiosystems DT-24 Thermal Cycling PCR System). Plates are then read on a fluorescent plate reader (e.g. CLARIOstar Plus Plate Reader (BMG)) and scored automatically. Individuals are notified of the test results by SMS or email. In the case of positive tests, the local heath authority is also informed. Throughput is mostly limited by the RT-PCR capacity, and can reach > 600,000 samples per day for a single 100 × 384 well plate water bath PCR system.

The PCR testing described is EU-wide CE approved in gargle- and smear-based versions and is conducted in accordance with relevant guidelines and regulations and with appropriate informed consent from tested individuals.

### Mathematical modelling

The methods used to construct the model are described in Ref.^26^. The model is based on the established Kermack–McKendrick theory, adjusted to COVID-19 and implemented as Mathematica 12 notebooks. In the model, the effect of population-wide testing of specific fractions of the population was superimposed on the effects of the series of social distancing/lockdown measures taken or the social distancing/lockdown measures in force at the assumed beginning of the tests (15th of October 2020). In predicting the later development of the pandemic, both the effects of new variants (UK variant from January 2021) and vaccination (also from the beginning of the current year) are taken into account. Access the three notebooks used in this work here: http://www2.math.uni-wuppertal.de/~scholz/model-epidem.html.

## Data Availability

The mathematical used in this work is based on the established Kermack–McKendrick theory, adjusted to COVID-19 and implemented as Mathematica 12 notebooks. The three notebooks used in this work are available for download through http://www2.math.uni-wuppertal.de/~scholz/model-epidem.html.
